# Integrating Health Data-Driven Machine Learning Algorithms to Evaluate Risk Factors of Early Stage Hypertension at Different Levels of HDL and LDL Cholesterol

**DOI:** 10.3390/diagnostics12081965

**Published:** 2022-08-14

**Authors:** Pen-Chih Liao, Ming-Shu Chen, Mao-Jhen Jhou, Tsan-Chi Chen, Chih-Te Yang, Chi-Jie Lu

**Affiliations:** 1 Division of Cardiology, Cardiovascular Center, Far Eastern Memorial Hospital, New Taipei City 220, Taiwan; 2 Department of Healthcare Administration, College of Healthcare and Management, Asia Eastern University of Science and Technology, New Taipei City 220, Taiwan; 3 Graduate Institute of Business Administration, Fu Jen Catholic University, New Taipei City 242, Taiwan; 4 Department of Medical Research, Far Eastern Memorial Hospital, New Taipei City 220, Taiwan; 5 Department of Business Administration, Tamkang University, New Taipei City 251, Taiwan; 6 Artificial Intelligence Development Center, Fu Jen Catholic University, New Taipei City 242, Taiwan; 7 Department of Information Management, Fu Jen Catholic University, New Taipei City 242, Taiwan

**Keywords:** health data-driven, high-density lipoprotein cholesterol (HDL-C), low-density lipoprotein cholesterol (LDL-C), hypertension, machine learning

## Abstract

Purpose: Cardiovascular disease (CVD) is a major worldwide health burden. As the risk factors of CVD, hypertension, and hyperlipidemia are most mentioned. Early stage hypertension in the population with dyslipidemia is an important public health hazard. This study was the application of data-driven machine learning (ML), demonstrating complex relationships between risk factors and outcomes and promising predictive performance with vast amounts of medical data, aimed to investigate the association between dyslipidemia and the incidence of early stage hypertension in a large cohort with normal blood pressure at baseline. Methods: This study analyzed annual health screening data for 71,108 people from 2005 to 2017, including data for 27 risk-related indicators, sourced from the MJ Group, a major health screening center in Taiwan. We used five machine learning (ML) methods—stochastic gradient boosting (SGB), multivariate adaptive regression splines (MARS), least absolute shrinkage and selection operator regression (Lasso), ridge regression (Ridge), and gradient boosting with categorical features support (CatBoost)—to develop a multi-stage ML algorithm-based prediction scheme and then evaluate important risk factors at the early stage of hypertension, especially for groups with high-density lipoprotein cholesterol (HDL-C) and low-density lipoprotein cholesterol (LDL-C) levels within or out of the reference range. Results: Age, body mass index, waist circumference, waist-to-hip ratio, fasting plasma glucose, and C-reactive protein (CRP) were associated with hypertension. The hemoglobin level was also a positive contributor to blood pressure elevation and it appeared among the top three important risk factors in all LDL-C/HDL-C groups; therefore, these variables may be important in affecting blood pressure in the early stage of hypertension. A residual contribution to blood pressure elevation was found in groups with increased LDL-C. This suggests that LDL-C levels are associated with CPR levels, and that the LDL-C level may be an important factor for predicting the development of hypertension. Conclusion: The five prediction models provided similar classifications of risk factors. The results of this study show that an increase in LDL-C is more important than the start of a drop in HDL-C in health screening of sub-healthy adults. The findings of this study should be of value to health awareness raising about hypertension and further discussion and follow-up research.

## 1. Introduction

Cardiovascular disease (CVD) is a major worldwide health burden today. Several large cohort studies, including the Framingham Heart Study, demonstrate that hypertension and dyslipidemia (high LDL cholesterol (LDL-C) and low HDL cholesterol (HDL-C)) are important risk factors of future CVD [1]. A systemic review and meta-analysis suggest that the lowering of blood pressure to the normotensive range should be considered for the prevention of CVD [2]. Hypertension and dyslipidemia have an additive effect on the incidence of coronary heart disease in subjects with both conditions compared to those with only one or the other [3]. In modern management of coronary heart disease and cerebrovascular disease, the lowering of both blood pressure and LDL-C is important [4,5,6].

In recent years, the definition of normotensive has become more stringent (less than 130/85 mmHg) [7], because raised blood pressure is the leading cause of death globally [8]. Several studies have found an association between CVD and early stage hypertension [9,10,11]. In addition, there is a positive association between abnormal serum cholesterol levels and hypertension [12,13]. It is noteworthy that early prediction of hypertension is an important issue for individuals with dyslipidemia. However, the relationship between early stage hypertension (or prehypertension) and dyslipidemia remains unclear [14]. The medication of blood statin effect pressure-lowering has been confirmed by a meta-analysis published paper [15], which showed a small reduction (−2.62 mmHg) in systolic blood pressure (95% CI: −3.41 to −1.84; *p* < 0.001). In addition, Borghi et al. [16] also found that better control of LDL-C is associated with lower antihypertensive treatment in a large cohort study. These studies suggested that LDL-C is associated with hypertension and early stage hypertension. The association between HDL-C and hypertension is poorly understood. The Framingham Heart Study considered HDL-C to have a cardio-protective effect. The relationship between HDL-C and blood pressure is less clear. A positive linear relationship has been reported [17], but some reports have shown a slightly U-shaped relationship, or an inverted J-shaped relationship [18,19].

As the risk factors of CVD, both hypertension and dyslipidemia are mostly mentioned. Blood pressure is an important leading indicator of health hazards, especially for CVD or other related chronic diseases. Predicting the presence of early stage hypertension could be provided the possibility to prevent future CVD or chronic diseases. As mentioned above, the result of most studies demonstrated the correlation between BP and either LDL-C or HDL-C. It means that there is a potential relationship between BP and dyslipidemia. Dyslipidemia and high LDL-C and/or low HDL-C are associated with atherosclerosis and could lead to a change in blood pressure. This study aimed to investigate the association between dyslipidemia and the incidence of early stage hypertension in a large cohort with normal blood pressure at baseline.

The application of data-driven machine learning (ML) algorithms to the analysis of healthcare data and/or medical records is not uncommon, and there is even an increasing trend in publications introducing artificial intelligence technology [20,21,22,23]. The advantages of ML algorithms include the effective investigation of complex relationships between risk factors and outcomes, and promising predictive performance with vast amounts of medical data [22,23,24,25,26]. Our study used five ML techniques—stochastic gradient boosting (SGB), multivariate adaptive regression splines (MARS), least absolute shrinkage and selection operator logistic regression (Lasso), ridge logistic regression (Ridge), and gradient boosting with categorical features support (CatBoost)—to develop a multi-stage ML algorithm-based prediction scheme.

SGB is a model creates multiple additive regression trees with the decision tree algorithm by combining bagging and boosting techniques [27]. MARS is a nonlinear spline regression and a non-parametric form of the regression analysis algorithm [28]. Lasso and Ridge are both improved conventional logistic regression models using shrinkage regularization techniques [29]. CatBoost is an algorithm of integrating gradient boosting and multiple categorical variables based on gradient boosting decision tree framework. [30]. These five ML methods have been widely used in various healthcare and/or medical informatics applications [31,32,33,34,35,36,37,38,39,40] as they could generate more effective predictive models than classical logistic regression model. They have also successfully been applied the field of predicting hypertension [41,42,43,44]. For example, Chang et al. [41] constructed a multiple predictive model for hypertension and hyperlipidemia using MARS. Lee et al. [38] used CatBoost method to predict intracranial hypertension and arterial blood pressure in patients with acute phase traumatic brain injury. Ang et al. [42] applied Lasso method to predict non-contact hypertension by the facial characteristics data of subjects. Shan et al. [43] utilized the ridge method to evaluate intracranial hypertension in traumatic brain injury patient. Chai et al. [44] used SGB and CatBoost methods to develop adolescent hypertension prediction model based on anthropometric measurements data. This study aimed to investigate the association between dyslipidemia and the incidence of early stage hypertension in a large cohort with normal blood pressure at baseline. The proposed scheme was used for each of four subgroups grouped by HDL-C and LDL-C criteria to predict early stage hypertension, evaluate relatively important risk factors, and then integrate feature selection results.

## 2. Materials and Methods

### 2.1. Data

The subjects of this study were data tracked continuously for a long time in Taiwan. It belongs to the annual health examination data of sub-health groups. The data is of excellent quality and dozens of international journal papers have been published successively. In this study, health screening was applied to the data of sub-healthy adults. The research results are more helpful to provide government health units with policy directions for preventive population health and health promotion. The data sets used were sourced from the MJ Group (Taipei, Taiwan)—a major health screening center in Taiwan—for the years 2005 to 2017. Many studies from Taiwan published in international journals have used the MJ Health Checkup-Based Population Database (MJPD) and are collated in http://www.mjhrf.org/main/page/resource/en/#resource07 (accessed on 18 April 2022). These include studies on metabolic syndrome [45,46,47] and chronic kidney disease [24,48]. The MJPD database includes data collected from four MJ clinics that carry out periodic health examinations of the center’s approximately 71,000 members. The database can be accessed by academic researchers on request. All the data sets used in this study were authorized by and received from the MJ Health Research Foundation (Approval No.: MJHRF-2016005A). The data application procedures are described at http://www.mjhrf.org/main/page/release1/en/#release01 (accessed on 18 April 2022). In the case of ethical issues regarding the use of data in the database, the protocol of this study was evaluated and deemed acceptable by the Research Ethics Review Committee of Far Eastern Memorial Hospital (FEMH-IRB-107127-E, Protocol Version1, 15 February 2022) and the MJ Health Research Foundation, and approved by ClinicalTrials.gov (ID: NCT05225454). The study was conducted according to the guidelines of the Declaration of Helsinki and fulfilled the Institutional Review Board ethics requirements by anonymizing all data before analysis.

**Figure 1:**
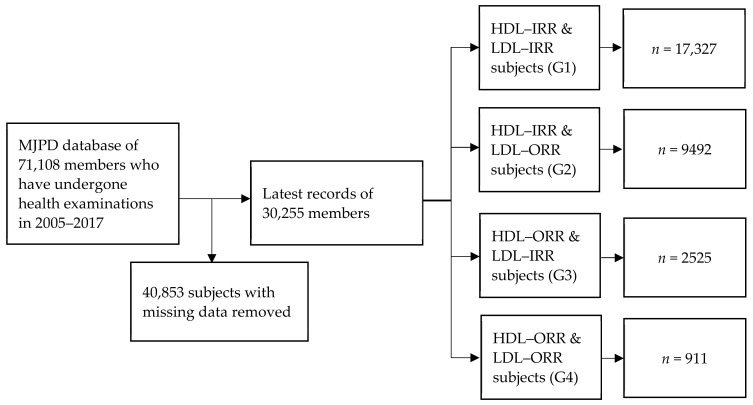
The enrollment flowchart for subject identification.

Figure 1 shows the subject identification process of this study. The data consisted of the health examination indices and questionnaire records of the 71,108 members in the MJPD database from 2005 to 2017. Table 1 shows the 27 health examination indices and questionnaire variables developed in this study. Because every member may have multiple records, only the latest records were analyzed for subjects who had undergone multiple health examinations. In all, 40,853 subjects were removed because they had missing data for certain variables. After data processing, 30,255 eligible subjects remained. Table 2 shows the demographics and statistical analysis of subjects’ characteristics.

Using the HDL-C and LDL-C reference ranges of the American Heart Association as baseline values, the data were then categorized into four subgroups based on whether the HDL-C and LDL-C values were within the reference range (IRR) or out of the reference range (ORR). An irregular HDL value was identified based on the reference range for the individual’s gender. The ORR values of HDL-C for men and women are more than 40 and 50 mg/dL, respectively. The ORR value of LDL is below 130 mg/dL. The four subgroups analyzed were as follows: 17327 HDL–IRR and LDL–IRR subjects (G1); 9492 HDL–IRR and LDL–ORR subjects (G2); 2525 HDL–ORR and LDL–IRR subjects (G3); and 911 HDL–ORR and LDL–ORR subjects (G4).

Past studies have not investigated whether the HDL/LDL indicators are normal or not divided into four groups, and have not applied the multivariate and different algorithms of machine learning tools.

### 2.2. Proposed Multi-Stage Machine Learning Algorithm-Based Scheme

**Figure 2:**
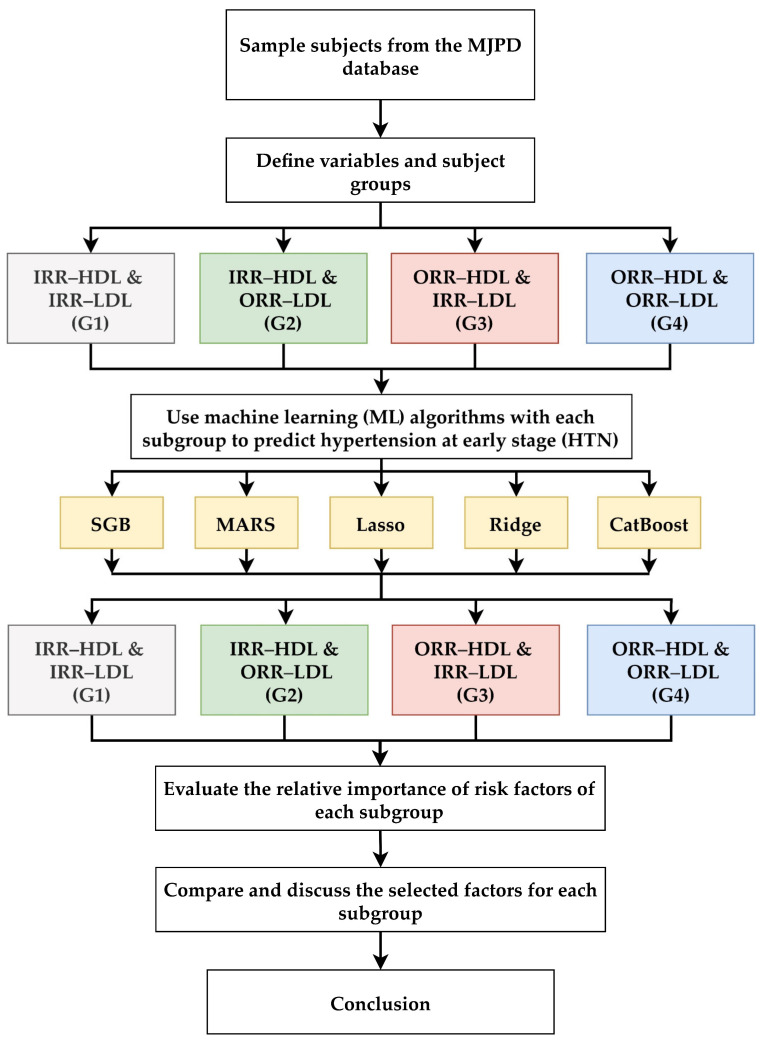
Proposed multi-stage machine learning algorithm-based scheme.

This study developed a multi-stage hypertension prediction framework based on the machine learning algorithms for the four subgroups (G1, G2, G3, and G4) to identify, integrate, and examine the key risk factors for hypertension prediction in each subgroup. The overall procedure of the multi-stage machine learning algorithm-based scheme is shown in Figure 2. In the prediction framework, the first step was to collect the subjects’ health examination data from the MJPD database for analysis. The second step was to define the risk variables, identify subjects, and distinguish between the subjects in the four subgroups. The third step was to use the five learning algorithms (SGB, MARS, Lasso, Ridge, and CatBoost) to develop the prediction model for each subgroup in Table 1 using 24 risk factors as predictor variables (excluding HDL-C, LDL-C, and HTN) and the HTN as the target variable.

SGB implementation process sequentially generates many decision trees that are weak learners through multiple iterations so that each tree is trained based on the residual of the previous iteration [27,49]. The iterative process continues until the guideline of the maximum number of iterations or the convergence condition is reached. Finally, the cumulative results of many trees are obtained by weighed summation, and then the final robust model is determined.

MARS uses multiple piecewise linear segments (splines) with differing gradients. Its concept considers each sample as a knot and divides it into several sections for successive linear regression of the data within each section [28]. In the process for determining knots, a forward algorithm is used to select all possible basic functions and their corresponding knots, and a backward algorithm eliminates all basic functions to generate the best combinations of existing knots.

The Lasso Ridge methods share the same basic concept. The Lasso principle integrates the least absolute selection and shrinkage operator with L1 regularization, which can force compression of the coefficients of covariates making a minor contribution to the model to exactly zero to attain lower variance to reduce the problem of overfitting [50,51]. The main difference is the use of the L2 regularization technique to shrink model coefficients in Ridge. L2 regularization does not eliminate the coefficients or encourage sparse models. The addition of appropriate L2 penalties to the model shrinks all the coefficients to a nonzero value or a value approaching zero, and then minimizes the sum of squared error, and further controls the trade-off between bias and variance to reduce overfitting [52].

The CatBoost process is constructed using random multiple permutations generated to obtain gradients and correlations with the category variable [30]. As decision trees are weak learners, gradient boosting is successively fitted to each decision tree, where each tree is developed with a smaller loss compared to the previous one. Finally, it integrates all combinations and classification variables of the current tree into a sequence to generate the final model. CatBoost uses the ordered method of gradient boosting, which overcomes the prediction shift of the gradient estimation, and thus improves the algorithm’s accuracy and generalization [30,53].

When constructing each model, the data set was randomly partitioned 80% for the training data set and 20% for the testing data set. Model hyperparameter tuning and validation were executed using a 10-fold cross-validation approach on all the samples available in the training data set. The model with the best hyperparameter was chosen as the final model. The predictive performance of the models was assessed using the following measures: sensitivity, specificity, and area under the receiver operating characteristic (ROC) curve (AUC). However, the models were highly influenced by data class distribution of these measures. Therefore, we also computed balanced accuracy (BA) and g-mean (GM) because they can be excellent measures for evaluating skewed data in any data class [54,55,56,57].

The used five ML models were implemented using R software of version 3.6.2 and RStudio software of version 1.1.453 (http://www.R-project.org; accessed on 25 May 2022; https://www.rstudio.com/products/rstudio/; accessed on 25 May 2022). Each algorithm was performed based on the related R packages. SGB, MARS, Lasso, Ridge, and CatBoost were implemented in “gbm” R package version 2.1.8 [58], “earth” R package version 5.3.1 [59], “glmnet” R package version 4.1-4 [60], and “catboost” R package version 1.0.6 [61], respectively. For developing efficient and exact SGB, MARS, Lasso, Ridge, and CatBoost models, the “caret” R package version 6.0-92 was used for all models to estimate the best hyperparameters [62].

In the fifth step, after obtaining the effective prediction models for all four subgroups as derived by SGB, MARS, Lasso, Ridge, and CatBoost, the relative importance of variables generated by each algorithm for each risk factor was also obtained. The variable importance of the most and least important risk factors was 100 and 0, respectively. Values can be repeated, that is, two or more variables can have similar variable importance. Because different machine learning algorithms have different model development principles and features, the variable importance values generated by the five algorithms for a single risk factor can differ. Within the same subgroup, a single robust and complete value for variable importance can be generated for each risk factor in order to facilitate subsequent comparison of variable rankings and identification of important risk factors. We generated a single consolidated value of variable importance based on the mean value of variable importance derived from the five machine learning models.

In the sixth step, we compared the important variables in G1 to G4 in order to examine and discuss their similarities and differences. The seventh and final step was to propose the conclusions of this study.

## 3. Results

**Figure 3:**
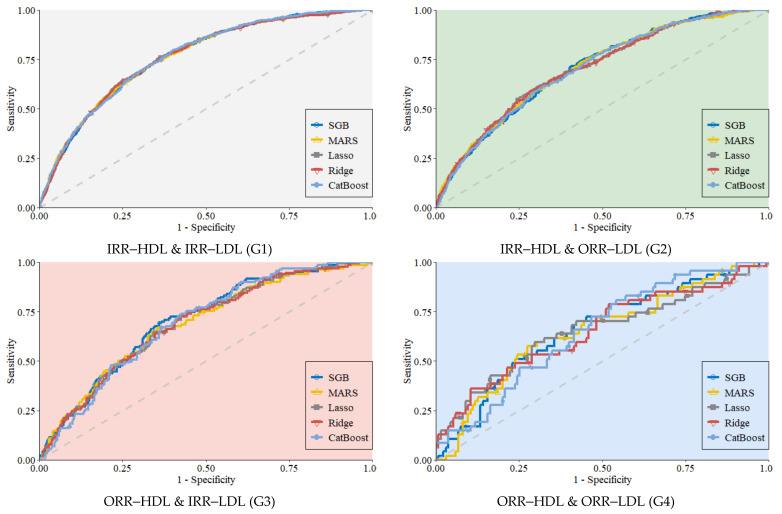
ROC curves of the five algorithms for each subgroup.

Table 3 presents the model prediction performance of SGB, MARS, Lasso, Ridge, and CatBoost in relation to the four subgroups (HDL–IRR & LDL–IRR [G1], HDL–IRR & LDL–ORR [G2], HDL–ORR & LDL–IRR [G3], and HDL–ORR & LDL–ORR [G4]). Figure 3 presents the subgroup performance of the five models using ROC curves. To compare the predictive performance of the five methods in each of four subgroups, the DeLong’s test was used in this study to compare AUC values between the five ML models. DeLong’s test is one of the useful methods to determine if there is a statistically significant difference between the performances of the methods based on AUC values [63]. Table 4 shows pairwise comparisons of AUC values of the five used ML methods in all subgroups using DeLong’s test. It can be observed that the performance of any two ML methods is not significant different as all *p*-values in the table are above 0.05. That is, the prediction performances of the models were similar for each of the four subgroups

However, the results differed between subgroups. For G1, the AUC of each algorithm was greater than 0.761 and was the highest among the subgroups. This shows that model prediction accuracy was highest for G1. Specifically, the Ridge algorithm had the highest sensitivity at 0.668, CatBoost had the highest specificity at 0.791 and the highest AUC at 0.764, while Lasso generated the highest values for BA and GM at 0.700 and 0.698, respectively. Lasso was comparatively the best prediction algorithm for G1.

For G2, the AUC of each algorithm was greater than 0.703 and was the second highest among the subgroups. Specifically, the Ridge algorithm had the highest sensitivity at 0.713, MARS had the highest specificity at 0.735 and the highest AUC at 0.707, SGB had a high BA at 0.655, and Lasso had the highest GM at 0.653. MARS was comparatively the best prediction algorithm for G2.

For G3, the AUC of each algorithm was greater than 0.68 and was the third highest among the subgroups. Specifically, the MARS algorithm had the highest sensitivity at 0.660, CatBoost the highest specificity at 0.741, and SGB the highest AUC, BA, and GM values at 0.702, 0.668, and 0.668, respectively. SGB was comparatively the best prediction algorithm for G3.

For G4, the AUC of each algorithm was greater than 0.649 and was the lowest among the subgroups. This shows that prediction of G4 was more difficult compared to the other subgroups. Specifically, the MARS algorithm had the highest sensitivity and BA values at 0728 and 0.651, respectively; CatBoost had the highest specificity at 0.809; SGB had the highest AUC at 0.658; and Lasso had the highest BA and GM values at 0.651 and 0.649, respectively. Lasso was comparatively the best prediction algorithm for G4.

In general, even though the overall prediction performance differed between subgroups, all five machine learning algorithms had promising and similar performance in hypertension prediction.

The variable importance generated by the five machine learning algorithms provides high reference value because of the similarity in prediction performance of the models. However, the variable importance of the same risk factor differed between algorithms. To account for the variable importance generated by every algorithm, we derived the mean importance of each risk factor based on the five variable importance values.

**Figure 4:**
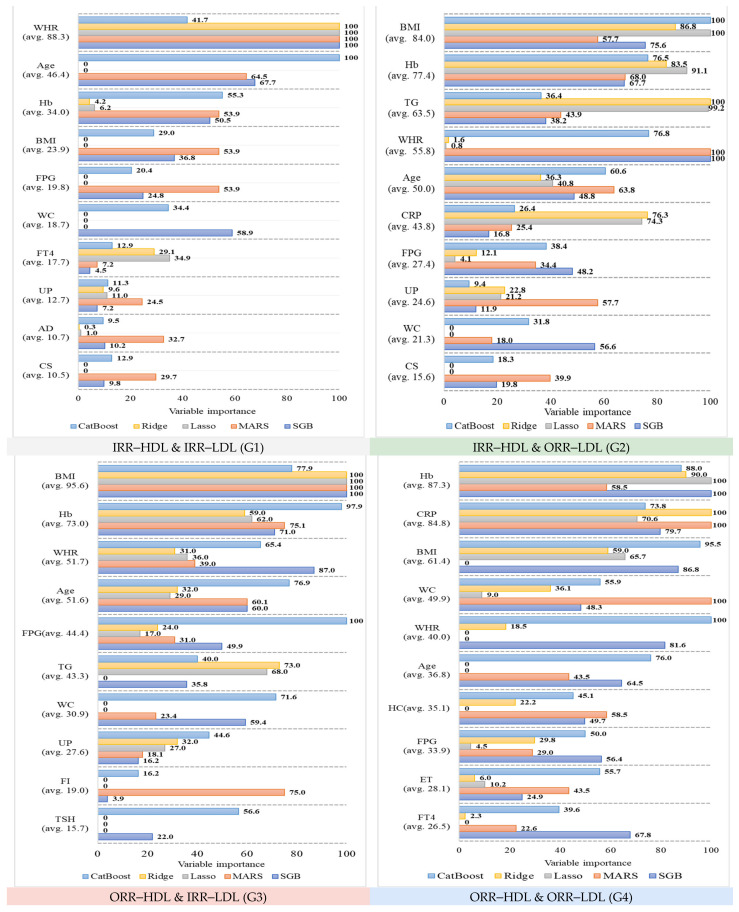
The variable importance generated by the generated by the five algorithms for each risk factor in the four subgroups.

Figure 4 shows the individual variable importance values generated by the five algorithms for each risk factor in the four subgroups. The 10 risk factors with the highest variable importance are presented for each subgroup in decreasing order of mean importance. For example, in G1, SGB, MARS, Ridge, and Lasso chose waist-to-hip ratio (WHR) as the most important variable, with an importance value of 100. On the other hand, CatBoost determined WHR to be of moderate importance at 41.7, although it is the most important risk factor in G1, with a mean importance of 88.3. Similarly, the second most important risk factor in G1 was age, with a mean variable importance of 46.4. Among the five models, age was chosen by CatBoost as the most important variable, with an importance value of 100, while SGB and MARS determined it to be of moderate importance at 67.7 and 64.5, respectively. However, Ridge and Lasso determined age to be the least important variable, with an importance of 0. In general, age remained the second most important variable in G1. Using the same concept and method, we were able to derive the ranking of variable importance for each of the four subgroups, as shown in Table 5.

Table 5 shows that the ranking of variable importance differs between subgroups. For example, the three most important variables (in decreasing order of mean variable importance) in G1 were WHR, age, and hemoglobin (Hb), whereas in G2 they were body mass index (BMI), Hb, and triglycerides (TG), in G3 they were BMI, Hb, and WHR, and in G4 they were Hb, C-reactive protein (CRP), and BMI. The similarities and differences between the 10 most important variables in the four subgroups will be elaborated in the Discussion section.

To distinguish between subgroups, the overall degree of similarity between the importance rankings of all 24 prediction variables in the four subgroups was represented by the correlation coefficients (*R*) of variable importance ranking, as shown in Table 6. The closer the *R* value was to 1, the more similar were the variable importance rankings of two subgroups and the more distant *R* was from 1, the less similar were the variable importance rankings of the subgroups.

The results of the analysis showed that the group whose LDL-C began to rise but whose HDL-C was still within the reference range (IRR–HDL & ORR–LDL [G2]) had a risk factor ranking similar to that of the group whose HDL-C began to decrease but whose LDL-C was still within the reference range (ORR–HDL & IRR–LDL [G3]), and the types of data were also similar (*R* = 0.899). However, in the comparison of G2 and G3 with the group whose HDL-C and LDL-C values were outside the reference range (ORR–HDL & ORR–LDL [G4]) shows that the correlation coefficient for G2 vs. G4 (*R* = 0.707) was higher than that for G3 vs. G4 (*R* = 0.602). This means that the group with abnormal LDL-C (G2) was more similar to the group with abnormal HDL-C and LDL-C (G4) than the group with abnormal HDL-C (G3); therefore, the beginning of abnormal LDL-C is a leading indicator and the start of the rise in LDL-C has reference value for prediction of prehypertension.

## 4. Discussion

This study identified risk factors that have utility in the prediction of hypertension in different dyslipidemia groups. We applied several predictive models using machine learning algorithms, and the results obtained with the different models were similar.

Hypertension is a worldwide health burden, with high prevalence in those with cardiovascular disease. According to the World Health Organization, about 17 million people die from CVD worldwide, and about 9.4 million die from hypertension. The prevalence of hypertension is about 29% worldwide and is expected to increase from 26% in 2000 to 29.2% in 2025. Several studies have advanced an association between early stage hypertension (or prehypertension) and CVD, but the relationship between blood pressure and mortality is controversial [64,65,66,67]. The inconsistent results may be related to the age of participants, associated metabolic risk factors (e.g., abnormal lipid profiles), and the definition of early stage hypertension (prehypertension). As there is a positive association between blood pressure and cardiovascular morbidity or mortality [2], the definition and management of hypertension should be more aggressive [4,5,6,7]. It is well known that positive associations between CVD and high blood pressure (BP) or dyslipidemia were identified as early as half a century ago in the Framingham Heart Study. Dyslipidemia, either an increase in LDL-C or a decrease in HDL-C, also plays an important role in the development of CVD. Meanwhile, the biological interrelation between hypertension and LDL-C [13,68] or HDL-C [12,69,70] has been documented. The structural and functional change in LDL-C and HDL-C, inflammation, and oxidative stress may be associated with vascular atherosclerotic processes, and lead to elevation of blood pressure [71].

The relationships between HDL-C concentration categories and blood pressure are U- or J-shaped [69,70,72,73,74]. In the Kanagawa Investigation of Total Checkup Data from the National Database-9 study, Nakajima et al. [70] found inverted J-shaped relationships between HDL-C and odds ratios for hypertension (≥140/90 mmHg) using the logistic regression analysis method, and both low and extremely high HDL-C concentrations are associated with high blood pressure within both sexes. In the South-West Seoul (SWS) Study, the elderly population with prehypertension combined with low HDL-C showed a twofold higher risk of all-cause mortality (HR: 2.01; 95% CI: 1.11–3.64) [69]. These studies showed that low HDL-C concentration is positively related to high blood pressure, but a linear relationship under extremely high HDL-C is not found. In clinical trials with cholesteryl ester transfer protein (CETP) inhibitors, a substantial increase in HDL-C concentration did not show a protective effect against CVD events. In addition, a slight increase in systolic blood pressure of 1.2–5.4 mmHg has been shown after intervention [72,73]. The tendency to develop hypertension is correlated with HDL-C subfraction (HDL-3 concentration) and total HDL-C concentration [74]. As mentioned above, it is difficult to predict early stage hypertension with only HDL-C. In health screening data for sub-healthy adults, the group in which HDL-C was beginning to decrease but LDL-C remained within the reference range was smaller than the group in which LDL-C was beginning to rise but HDL-C remained within the reference range. In this study (Table 6), we found that LDL-C is a leading indicator, and rising LDL-C is a reference for predicting prehypertension. This finding agrees with the results of previous studies that showed that the relationship between HDL-C concentration and blood pressure is U- or J-shaped.

It is basic knowledge that the higher the LDL-C level will increase the risk of developing cardiovascular disease (CVD). With intervention medical trials, using lipid-lowering agents to reduce LDL-C had shown consistent reductions in major CVDs [4,5,6]. Otsuka et al. [75] showed the development of hypertension according to LDL-C quintiles in Asian populations. Their results indicated the risk of hypertension was 1.27 times higher in the highest quintile compared to the lowest quintile [75]. Most previous studies identified a relationship between LDL-C and CVD, but really did not make sure the incidence of hypertension.

This is the first study demonstrating an association between dyslipidemia and the risk of incident hypertension. Otsuka et al. proposed several mechanisms for dyslipidemia and the increased risk of hypertension. First, dyslipidemia, may impair endothelial function and regulation of blood pressure by disrupting the production of nitric oxide. Second, by reducing baroreflex sensitivity, dyslipidemia may predispose individuals to the development of hypertension. Third, dyslipidemia decreases the distensibility of large elastic arteries. This decrease may reduce the wind vessel effect, and then increase systolic blood pressure. Fourth, a lack of physical activity or regular exercise and a high-fat daily diet promotes obesity. The adipose tissue excessively secretes adipocytokines, and the cytokines result in insulin resistance and subsequent activation of the sympathetic nervous system and the renin-angiotensin system in obese individuals. Those biological changes have been confirmed and reported to increase blood pressure and raise incident hypertension.

The presence of dyslipidemia in subjects with early stage hypertension (prehypertension) can significantly increase the risk of cardiovascular mortality [69]. Individuals with dyslipidemia and elevated blood pressure have metabolic syndrome. In previous studies, metabolic syndrome was associated with a higher risk of CVD mortality in middle-aged or elderly populations [76,77]. In subjects with type 2 diabetes, a target blood pressure of 120 mmHg, compared to 140 mmHg, did not reduce the rate of fatal and nonfatal CVD events in the ACCORD study [78]. However, in subjects without diabetes, the prognostic benefit of blood pressure control was clarified in the SPRINT trial [79]. In the same study, with a target SBP of less than 120 mm Hg, compared to less than 140 mm Hg, the results showed that in lower rates of fatal and nonfatal major cardiovascular events and all-cause death. During the follow-up period (median, 3.26 years) of this clinical trial, 25% of subjects showed a lower relative risk of cardiovascular-related outcomes, including the composite outcomes of myocardial infarction, stroke, acute coronary syndrome not resulting in acute or chronic myocardial infarction, acute decompensated heart failure, death from cardiovascular causes, etc. Additionally, the rates of lots other important outcomes in the treatment group, including death from cardiovascular causes (reduce 43% relative risk), heart failure (reduce 38% relative risk), and death from any cause (reduce 27% relative risk), was lower than the control group [79]. These results indicate that it is valuable to aggressively treat individuals with early stage hypertension. In addition, subjects with early stage hypertension and dyslipidemia are at a greater risk of mortality, suggesting that it is reasonable to treat this specific group to improve their prognosis.

Several factors (Table 5) are associated with early stage hypertension, including age, BMI, waist circumference (WC), Hb, CRP, etc. The correlation between hypertension and age, BMI, WC, or WHR is well known. The Hb level is also a positive contributor to blood pressure elevation and was one of the top three important risk factors in all four groups. In a large cohort study, Atsma et al. reported that systolic blood pressure increased by 0.7 mm Hg for every 0.9 mm Hg per millimole per liter increase in the hemoglobin level, and the results for diastolic blood pressure were comparable [80]. There was no gender difference in the study. Several mechanisms for the association between hemoglobin and blood pressure have been proposed. Hemoglobin is positively associated with pulse-wave velocity, an indicator of arterial stiffness, and increased systolic and diastolic blood pressure [81]. Nitroxide (NO), produced in the blood vessel endothelial cells, relaxes vascular smooth muscles, and thereby controls blood pressure. Acellular Hb may bind to NO and cause vessel constriction and elevation of blood pressure [82]. Increased Hb levels may lead to increased blood viscosity, and increased blood viscosity may worsen cardiovascular function, but the production of NO may also increase. In this study, blood viscosity was not measured; therefore, we do not know the influence of viscosity on blood pressure.

C-reactive protein is a biomarker of systemic inflammation. In hypertensive individuals, CRP levels are associated with cardiovascular events and end-organ damage because CRP is correlated with vascular stiffness and severity of atherosclerosis [83]. CRP appeared in the out-of-reference-range LDL-C groups (G2 and G4) and was one of the top two important variables in the ORR–HDL & ORR–LDL group. However, in normotensive individuals, genetic variability may influence circulating levels of CRP. A predictive association between changes in blood pressure and the development of hypertension remains controversial [83,84]. In this study, a residual contribution to blood pressure elevation is found in groups with increased LDL-C, which suggests that LDL-C levels are associated with CPR levels and that the LDL-C level may be a more important factor for predicting the development of hypertension.

Raised blood pressure is the leading cause of death globally [8]. The association between CVD and early stage hypertension is documented in several studies [9,10,11]. An individual with hypertension or dyslipidemia is predicted to be at lower risk for CVD than one with both of them. Predicting the probability of hypertension in dyslipidemia individuals with normal blood pressure is an important clinical issue. Because non-pharmacological methods, such as body weight control, aerobic exercise, salt restriction, and the DASH diet, are recommended to effectively prevent the development of hypertension. The machine learning model provided the possibility for early detection of the individual with early stage hypertension. In order to prevent future CVD, it would be valuable to suggest they modify their lifestyle aggressively. In addition, several other cofactors of early stage hypertension are also found in the ML model. It suggested that correcting those factors may be important for the development of hypertension. Meanwhile, the application of the ML model could be another method to establish a new direction for future studies to detect early stage hypertension. The implications of the model synthesized to clinical should be helpful and predictable in the public health practice.

The correlation between metabolic syndrome-related variables, including age, BMI, WC, WHR, fasting plasma glucose, and hypertension, is well known. The hemoglobin level is also a positive contributor to blood pressure elevation and it was one of the top three important factors in all four LDL-C/HDL-C groups in this study; therefore, it may be an important variable that affects blood pressure in the early stage of hypertension. A residual contribution to blood pressure elevation is found in groups with increased LDL-C. This suggests that LDL-C levels are associated with CPR levels, and that the LDL-C level may be a more important factor for predicting the development of hypertension. Even though this project is not a longitudinal study design, it may have confounding effects, but the cause of the huge amount of data, and the results were in line with clinical manifestations, so it still had application value in preventive medicine. Using directed acyclic graph to discuss the logical connective or confounding effects of the identified important risk factors is worth of further research.

## 5. Limitations

The main limitations of this study were the use of a single data set without comparing it to data from other countries and the lack of continuity of data analysis. In addition, this study was similar to previous studies that used cross-sectional data in that we estimated the influencing factors and speculated on the possible effects without providing a causal inference. To avoid the selected variables having logical connectors or confounding effects, the following research should be used the prospective or retrospective cohort study to prove the clinical significance. Another limitation of our study is that our inferences may not be suitable for outpatients or inpatients who are already ill. In addition, older patients, those with abnormal extreme values, or those using a physician’s prescription for an extended period were excluded from this study.

## 6. Conclusions

The five prediction models (SGB, MARS, Lasso, Ridge, and CatBoost) provided a similar classification of risk factors in this study. Based on the results of this study, we suggest that BMI, WHR, Hb, and CRP should be the important indicators of early stage hypertension in sub-healthy adults. A rise in the LDL-C level appears to be a signal and is more important than the start of a decrease in HDL-C. Raising awareness of hypertension is crucial in government health promotion activities, and the findings of this study should be of value for further discussions and follow-up research.

## Figures and Tables

**Table 1:** Characteristics or laboratory indices of participants for predicting early stage hypertension.

Abbreviation	Variable (Unit)	Description/Reference Range (RR)
SEX	Gender (sex)	(1) Male; (2) Female
Age	Age (y/o)	Number; Years old (y/o)
MS	Marital status	(1) Single; (2) Married, remarried, cohabiting; (3) Divorced; (4) Widowed
EL	Education level	(1) No formal education; (2) Elementary school; (3) Secondary school; (4) High school; (5) College; (6) University; (7) Graduate school
FI	Family income (NTD)	(1) Unwaged; (2) ≤200,000; (3) 200,001–400,000; (4) 400,001–800,000; (5) 800,001–1,200,000; (6) 1,200,001–1,600,000; (7) 1,600,001–2,000,000; (8) >2,000,000
BMI	Body mass index (kg/m^2^)	Number; Body weight/Body height^2^
BF	Body fat (%)	Number; Data collection from ©OMRON: HBF–702t
WC	Waist circumference (cm)	Number; WC measured with a tape measure by SOP.
HC	Hip circumference (cm)	Number; HC measured with a tape measure by SOP.
WHR	Waist-to-hip ratio (%)	Number; Waist circumference/Hip Circumference
Hb	Hemoglobin (g/dl)	Number; RR: Male: 13.5 < Hb < 17.5; Female: 12.0 < Hb < 16.0
FPG	Fasting plasma glucose (mg/dL)	Number; RR: 70 < FPG < 100
TG	Triglycerides (mg/dL)	Number; RR: TG ≤ 150
T-Cho	Total cholesterol (mg/dL)	Number; RR: 130 < T-Cho < 200
FT4	Free thyroxine 4 (ng/dL)	Number; RR: 0.70 < FT4 < 1.48
TSH	Thyroid-stimulating hormone (μIU/mL)	Number; RR: 0.47 < TSH < 5.00
CRP	C-reactive protein (mg/dL)	Number, RR: CRP < 0.5
UP	Urine protein	Qualitative test; (1) none (2) trace (+/−) (3) + (4) ++ (5) +++ (6) ++++
CS	Current smoker	(1) Never; (2) Passive smoking; (3) Quit; (4) Occasional; (5) Addicted
AD	Alcohol drinker	(1) Never; (2) Quit; (3) 1–2 times a week; (4) 3–4 times a week; (5) 5–6 times a week; (6) Addicted
CBN	Chews betel nut (*Areca catechu*)	(1) Never; (2) Quit; (3) 1–3 times a week; (4) 4–5 times a week; (5) Addicted
MB	Mealtime behavior	(0) Irregular; (1) Regular
ET	Excise time (hours)	Time spent exercising in the past two weeks. (1) <0.5; (2) 0.5–1; (3) 1–2; (4) >2
ST	Sleep time (hours)	Average sleeping time at night. (1) <4; (2) 4–6; (3) 6–7; (4) 7–8; (5) 8–9; (6) >9
HDL-C	High-density lipoprotein cholesterol (mg/dL)	Number; RR: Male: HDL-C > 40; Female: HDL-C > 50. IRR–HDL and/or ORR–HDL: the different RR values for males and females were considered.
LDL-C	Low-density lipoprotein cholesterol (mg/dL)	Number, RR: LDL-C < 130
HTN	Hypertension in early stage # SBP: Systolic blood pressure (mmHg) DBP: Diastolic blood pressure (mmHg)	(0) Normal subjects: SBP < 120 and DBP < 80 (1) HTN subjects: SBP ≥ 120 and DBP ≥ 80

Note: The laboratory data in the subject databases were obtained using the same biochemical examination apparatus (an automatic biochemical analyzer was provided by Hitachi Medical Device Co., Ltd., ©Hitachi-7600, Tokyo, Japan). HDL: IRR and/or ORR, meaning data were within the reference range (IRR) and/or data were out of the reference range (ORR). #: Hypertension in the early stage, the criterion refers to the guidelines of the American Heart Association (AHA).

**Table 2:** The demographics statistical analysis of subjects’ characteristics.

Ordinal Variable (Unit)		*N* (%)	Ordinal Variable (Unit)		*N* (%)
Gender	Male	15,628 (51.65%)	Chews betel nut (*Areca catechu*)	Never	28,784 (95.14%)
	Female	14,627 (48.35%)		Quit	1053 (3.48%)
Marital status	Single	4906 (16.22%)		1–3 times a week	264 (0.87%)
	Married, remarried, cohabiting	22,948 (75.85%)		4–5 times a week	50 (0.17%)
	Divorced	1144 (3.78%)		Addicted	104 (0.34%)
	Widowed	1257 (4.15%)	Mealtime behavior	Irregular	8384 (27.71%)
Education level	No formal education	438 (1.45%)		Regular	21,871 (72.29%)
	Elementary school	1958 (6.47%)	Excise time (hours)	<0.5	8361 (27.64%)
	Secondary school	1251 (4.13%)		0.5–1	13,513 (44.66%)
	High school	5655 (18.69%)		1–2	6409 (21.18%)
	College	6394 (21.13%)		>2	1972 (6.52%)
	University	9362 (30.94%)	Sleep time (hours)	<4	471 (1.56%)
	Graduate school	5197 (17.18%)		4–6	7375 (24.38%)
Family income (NTD)	Unwaged	1787 (5.91%)		6–7	14,787 (48.87%)
	≤200,000	2878 (9.51%)		7–8	6499 (21.48%)
	200,001–400,000	NA		8–9	NA
	400,001–800,000	6950 (22.97%)		>9	NA
	800,001–1,200,000	8256 (27.29%)	**Interval Variable (Unit)**		**Mean ± SD**
	1,200,001–1,600,000	4008 (13.25%)	Age (y/o)		47.25 ± 12.41
	1,600,001–2,000,000	2601 (8.60%)	Body mass index (kg/m^2^)		23.66 ± 3.59
	>2,000,000	3775 (12.48%)	Body fat (%)		26.76 ± 6.86
Urine protein	none	29,364 (97.06%)	Waist circumference (cm)		78.84 ± 10.17
	trace (+/−)	521 (1.72%)	Hip circumference (cm)		95.37 ± 6.31
	+	254 (0.84%)	Waist-to-hip ratio (%)		0.83 ± 0.08
	++	87 (0.29%)	Hemoglobin (g/dL)		14.14 ± 1.51
	+++	29 (0.10%)	Fasting plasma glucose (mg/dL)		103.2 ± 19.35
	++++	NA	Triglycerides (mg/dL)		115.79 ± 89.01
Current smoker	Never	22,339 (73.84%)	Total cholesterol (mg/dL)		196.99 ± 34.40
	Passive smoking	1066 (3.52%)	Free thyroxine 4 (ng/dL)		1.08 ± 0.15
	Quit	2450 (8.10%)	Thyroid-stimulating hormone (μIU/mL)		1.73 ± 1.77
	Occasional	1062 (3.51%)	C-reactive protein (mg/dL)		0.21 ± 0.39
	Addicted	3338 (11.03%)			
Alcohol drinker	Never	24,832 (82.08%)	**Control Variable (Unit)**		**Mean ± SD**
	Quit	650 (2.15%)	High-density lipoprotein cholesterol (mg/dL)		59.01 ± 14.92
	1–2 times a week	3225 (10.66%)	Low-density lipoprotein cholesterol (mg/dL)		118.77 ± 32.2
	3–4 times a week	1045 (3.45%)	**Dependent Variable (Unit)**		***N* (%)**
	5–6 times a week	NA	Hypertension in early stage (HTN)	SBP < 120 and DBP < 80	23,180 (76.62%)
	Addicted	503 (1.66%)		SBP ≥ 120 and DBP ≥ 80	7075 (23.38%)

**Table 3:** Model performance in predicting hypertension for HDL and LDL IRR/ORR subgroups.

Subgroup, Total *N* = 30,255	Method	Sensitivity	Specificity	AUC	BA	GM
IRR–HDL & IRR–LDL (G1) *n* = 17,327 (57.27%)	SGB	0.625	0.770	0.762	0.698	0.694
	MARS	0.659	0.732	0.762	0.695	0.694
	Lasso	0.645	0.755	0.762	0.700	0.698
	Ridge	0.668	0.725	0.761	0.696	0.696
	CatBoost	0.605	0.791	0.764	0.698	0.692
IRR–HDL & ORR–LDL (G2) *n* = 9492 (31.37%)	SGB	0.595	0.715	0.705	0.655	0.652
	MARS	0.567	0.735	0.707	0.651	0.645
	Lasso	0.691	0.617	0.705	0.654	0.653
	Ridge	0.713	0.594	0.705	0.653	0.650
	CatBoost	0.682	0.613	0.703	0.648	0.647
ORR–HDL & IRR–LDL (G3) *n* = 2525 (8.35%)	SGB	0.642	0.695	0.702	0.668	0.668
	MARS	0.660	0.649	0.685	0.655	0.655
	Lasso	0.572	0.733	0.688	0.653	0.648
	Ridge	0.583	0.718	0.687	0.650	0.647
	CatBoost	0.575	0.741	0.693	0.658	0.652
ORR–HDL & ORR–LDL (G4) *n* = 911 (3.01%)	SGB	0.581	0.702	0.658	0.642	0.639
	MARS	0.728	0.575	0.649	0.651	0.647
	Lasso	0.706	0.596	0.653	0.651	0.649
	Ridge	0.478	0.787	0.653	0.633	0.613
	CatBoost	0.456	0.809	0.650	0.632	0.607

Note: SGB: stochastic gradient boosting; MARS: multivariate adaptive regression splines; Lasso: least absolute shrinkage and selection operator; Ridge: ridge regression; CatBoost: gradient boosting with categorical features support. IRR–HDL & IRR–LDL: subjects whose HDL and LDL data were within the reference range; IRR–HDL & ORR–LDL: subjects whose HDL data were within the reference range and LDL data were out of the reference range; ORR–HDL & IRR–LDL: subjects whose HDL data were out of the reference range and LDL data were within the reference range; ORR–HDL & ORR–LDL: subjects whose HDL and LDL data were both out of the reference range.

**Table 4:** Pairwise comparisons of AUC values of the five used ML methods in all subgroup using DeLong’s test.

Subgroup	Methods	SGB	MARS	Lasso	Ridge
IRR–HDL & IRR–LDL (G1)	SGB	–			
	MARS	0.467	–		
	Lasso	0.286	0.716	–	
	Ridge	0.164	0.517	0.085	–
	CatBoost	0.068	0.350	0.647	0.912
IRR–HDL & ORR–LDL (G2)	SGB	–			
	MARS	0.643	–		
	Lasso	0.874	0.778	–	
	Ridge	0.957	0.711	0.494	–
	CatBoost	0.589	0.410	0.588	0.664
ORR–HDL & IRR–LDL (G3)	SGB	–			
	MARS	0.273	–		
	Lasso	0.319	0.857	–	
	Ridge	0.288	0.933	0.477	–
	CatBoost	0.436	0.653	0.742	0.933
ORR–HDL & ORR–LDL (G4)	SGB	–			
	MARS	0.774	–		
	Lasso	0.899	0.904	–	
	Ridge	0.906	0.910	0.992	–
	CatBoost	0.865	0.967	0.960	0.957

Note: The numbers in table are the corresponding *p*-values.

**Table 5:** Ranking of the top ten most important variables of the four subgroups.

Rank\Subgroup	IRR–HDL & IRR–LDL (G1)	IRR–HDL & ORR–LDL(G2)	ORR–HDL & IRR–LDL (G3)	ORR–HDL & ORR–LDL (G4)
1	WHR	BMI	BMI	Hb
2	Age	Hb	Hb	CRP
3	Hb	TG	WHR	BMI
4	BMI	WHR	Age	WC
5	FPG	Age	FPG	WHR
6	WC	CRP	TG	Age
7	FT4	FPG	WC	HC
8	UP	UP	UP	FPG
9	AD	WC	FI	ET
10	CS	CS	TSH	FT4

Note: AD: alcohol drinker; BMI: body mass index; CRP: C-reactive protein; CS: current smoker; ET: excise time; FI: family income; FPG: fasting plasma glucose; FT4: free thyroxine 4; Hb: hemoglobin; HC: hip circumference; TG: triglycerides; TSH: thyroid-stimulating hormone; UP: urine protein; WC: waist circumference; WHR: waist-to-hip ratio. Note: G1: group whose LDL-C and HDL-C were all within the reference range; G2: group whose LDL-C started to rise but HDL-C was still within the reference range; G3: group whose HDL-C started to decrease but LDL-C was still within the reference range; G4: group whose HDL-C and LDL-C values were out of the reference range.

**Table 6:** Correlation coefficients (*R*) of the variable importance ranking orders of the four groups.

Subgroup	IRR–HDL & IRR–LDL (G1)	IRR–HDL & ORR–LDL(G2)	ORR–HDL & IRR–LDL (G3)	ORR–HDL & ORR–LDL (G4)
IRR–HDL & IRR–LDL (G1)	1			
IRR–HDL & ORR–LDL (G2)	0.622	1		
ORR–HDL & IRR–LDL (G3)	0.633	0.899	1	
ORR–HDL & ORR–LDL (G4)	0.371	0.707	0.602	1

Note: G1: group whose LDL-C and HDL-C were all within the reference range; G2: group whose LDL-C started to rise but HDL-C was still within the reference range; G3: group whose HDL-C started to decrease but LDL-C was still within the reference range; G4: group whose HDL-C and LDL-C values were out of the reference range.

## Data Availability

Authorization is required for the use of all data sets collected from the MJ Health Research Foundation. The application procedures are accessed via this link. http://www.mjhrf.org/main/page/release1/en/#release01 (accessed on 18 April 2022).
